# The Wearable Activity Tracker Checklist for Healthcare (WATCH): a 12-point guide for the implementation of wearable activity trackers in healthcare

**DOI:** 10.1186/s12966-024-01567-w

**Published:** 2024-03-13

**Authors:** Kimberley Szeto, John Arnold, Carol Maher

**Affiliations:** Alliance for Research in Exercise Nutrition and Activity (ARENA), Allied Health and Human Performance, University of South Australia, North Terrace, GPO Box 2471, 5001 Adelaide, SA Australia

**Keywords:** Wearable activity tracker, Healthcare, Physical activity, Sedentary behaviour

## Abstract

Increasing physical activity in patients offers dual benefits, fostering improved patient health and recovery, while also bolstering healthcare system efficiency by minimizing costs related to extended hospital stays, complications, and readmissions. Wearable activity trackers offer valuable opportunities to enhance physical activity across various healthcare settings and among different patient groups. However, their integration into healthcare faces multiple implementation challenges related to the devices themselves, patients, clinicians, and systemic factors. This article presents the Wearable Activity Tracker Checklist for Healthcare (WATCH), which was recently developed through an international Delphi study. The WATCH provides a comprehensive framework for implementation and evaluation of wearable activity trackers in healthcare. It covers the purpose and setting for usage; patient, provider, and support personnel roles; selection of relevant metrics; device specifications; procedural steps for issuance and maintenance; data management; timelines; necessary adaptations for specific scenarios; and essential resources (such as education and training) for effective implementation. The WATCH is designed to support the implementation of wearable activity trackers across a wide range of healthcare populations and settings, and in those with varied levels of experience. The overarching goal is to support broader, sustained, and systematic use of wearable activity trackers in healthcare, therefore fostering enhanced physical activity promotion and improved patient outcomes.

**Supplementary Information:**

The online version contains supplementary material available at 10.1186/s12966-024-01567-w.

## Introduction

Physical activity is a cornerstone of health [1], and is particularly crucial for patients in hospital and other healthcare settings. Higher levels of physical activity during hospitalisation are associated with faster recovery [2–4], shorter length of hospital stay [2, 3], lower risk of early readmission [5], and lower rates of adverse events [4]. Low levels of lifestyle physical activity, are also correlated with increased rates of hospitalisation [6, 7], and higher overall healthcare costs [8]. Despite the overwhelming evidence supporting the benefits of physical activity for clinical populations [9], it remains under-promoted and under-prioritised in healthcare settings [10–12]. This gap between knowledge and practice not only exacerbates healthcare costs, but also contributes to the prevalence of a wide range of chronic diseases, costing the global economy an estimated INT$53.6 billion annually [13].

Barriers to physical activity promotion in healthcare are multi-faceted and varied. This ranges from individual patient and clinician factors, through to system-level factors such as policies and workplace structures that limit opportunities for promoting physical activity. As such, various strategies to improve physical activity promotion in healthcare are required [12], spanning interventions that target patient behaviour [14], through to large-scale policy implementation [15].

One behavioural approach to promoting physical activity in healthcare settings is by using wearable activity trackers. Wearable activity trackers are body-worn devices, often worn on the wrist, that can measure and allow users to track activity metrics like step count and physical activity minutes, and sometimes other outcomes like heart rate. They can be used to assess patient physical activity levels and enhance the delivery of physical activity interventions by supporting behaviour change strategies such as goal setting, self-monitoring, and behavioural feedback [16]. There is a substantial evidence base demonstrating that wearable activity trackers are effective for increasing physical activity across a wide range of healthy and clinical populations, age groups, and settings [17, 18].

Successful implementation of wearable activity trackers in healthcare settings requires a multifaceted approach, with co-ordination between technical, administrative, and clinical aspects [19–21]. From a technical standpoint, selecting an appropriate wearable activity tracker that provides relevant metrics and delivers a positive user experience is essential. Administratively, systems must be in place for loaning or accessing devices, creating user accounts, and tracking the return of devices. Clinically, protocols must be developed, and healthcare staff require training on using wearable activity trackers to support and encourage patients with their use for activity monitoring. Additional layers of complexity involve the establishment of documentation procedures and patient education materials, development of secure digital ecosystems for data storage and access, and the formulation of strategies to mitigate the added workload on healthcare staff related to device management [19, 46].

To bridge these gaps and take advantage of the potential of wearable activity trackers in promoting physical activity and improving patient outcomes, a structured and comprehensive approach to their implementation is required. This paper introduces the Wearable Activity Tracker Checklist for Healthcare (WATCH), a checklist that supports healthcare professionals and health service managers in developing procedures for using wearable activity trackers in healthcare settings. It comprises core elements to consider and plan for when developing procedures for using wearable activity trackers in different settings. Having procedures clearly outlined for using wearable activity trackers can help to address and mitigate some of the issues that may arise when implementing new innovations in healthcare. However, the wide range of populations and healthcare settings that wearable activity trackers can be used in means that procedures will differ across settings, and how wearable activity trackers are used to promote physical activity should be adapted to fit the intended context [22]. The WATCH provides a structured and comprehensive guide for developing procedures, while allowing the user to adapt details based on their specific circumstances.

## Background on checklist development

The WATCH was developed in consultation with a panel of experts and stakeholders in a Delphi study. The Delphi study involved four iterative online survey rounds spanning March 2021 to June 2022, and culminated in the identification of core elements for using wearable activity trackers in healthcare settings, and the development of the WATCH presented in this paper. The Delphi panel (*n* = 58) included healthcare professionals, researchers, and health service managers with experience or expertise on using wearable activity monitors in clinical settings. Rounds 1–3 identified the core elements to using wearable activity trackers in healthcare. These core elements along with the Consolidated Framework for Implementation Research (CFIR) [23] informed the development of a first draft of the WATCH. The draft checklist was presented to the Delphi panel in Round 4, with all 12 components reaching consensus as being very useful, and very clear and appropriate. A full report of the Delphi study is published in a companion paper [46].

## Aims and scope

In this user guide, we introduce the WATCH, a comprehensive checklist designed to support the integration of wearable activity trackers for physical activity promotion and monitoring in healthcare. Our primary aim is to provide an overview of how the WATCH can be used to develop and evaluate procedures for integrating wearable activity trackers in diverse healthcare settings and with various patient populations. The WATCH serves as a practical tool, directing users through the essential components they should consider when developing procedures. Importantly, while the WATCH provides a structured approach, it is designed to be adaptable to the needs of different contexts.

**Fig. 1:**
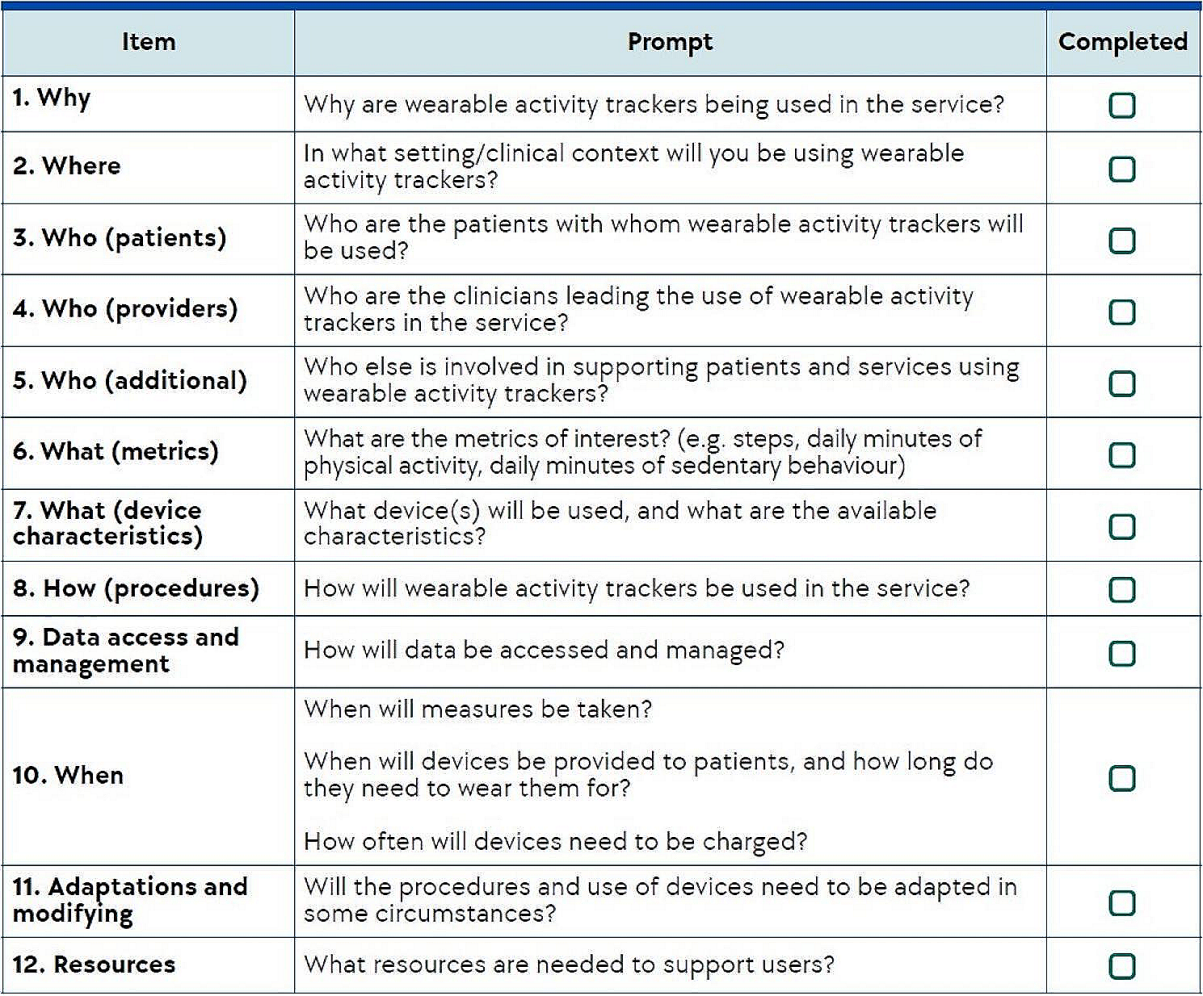
Short WATCH: 12-item checklist to guide wearable activity tracker implementation into healthcare

Users of the WATCH will include healthcare professionals, health service managers and researchers looking to leverage wearable activity trackers for physical activity promotion and monitoring in healthcare. The WATCH supports both the development and evaluation of projects that incorporate wearable activity trackers into healthcare. Each of the 12 items in the WATCH are described below with an accompanying explanation, and presented for ease of use in Fig. 1.

## WATCH items

### Item 1. Why?

Why will wearable activity trackers be used in the service?

*Identify the purpose(s) and goal(s).*

#### Explanation

The rationale, goals, and theory underpinning the use of wearable activity trackers should be clear. Outlining the purpose(s) and goal(s) for using wearable activity trackers will help differentiate essential elements from those that are optional. Reasons for using wearable activity trackers in healthcare settings may include:

- To assess and monitor daily activities (e.g. steps, sedentary time, light or moderate-to-vigorous physical activity, minutes of sleep, timing of activities such as bed time and wake time).
- Intervene on daily activities (e.g. steps, light activity, moderate-to-vigorous physical activity, sedentary behaviour, sleep).
- Potentially, monitor physiological parameters (e.g. heart rate).

Describing any underlying theories or behaviour change techniques can also assist in planning for the wearable activity trackers’ use (e.g. activity goal setting, prompts to move and break up sedentary behaviour, or feedback on sleep duration) [24].

### Item 2. Where?

In what setting/clinical context will you be using wearable activity trackers?

*Identify the health service setting, including location(s), nature of patient contact, and relevant infrastructure (e.g. internet connection, safe walking spaces).*

*Identify if previous efforts have been made to implement wearable activity trackers in the service.*

#### Explanation

Wearable activity trackers may be used in a wide range of healthcare settings and services [19]. Why and how they are used will vary depending on the needs of different settings and services. In settings with limited face-to-face contact between patients and clinicians, wearables can provide insights into patient activity for clinicians, and additional motivation and feedback for patients [25]. However, with less direct clinician involvement, patients and their family/carers may be required to take more responsibility for the device (e.g. charging, and donning after bathing). Outlining the characteristics of the setting can assist selection of suitable wearable activity trackers, and development of appropriate procedures. Additionally, identifying if wearable activity trackers have been used in the health service before (and what did and didn’t work) can help with the planning and conduct of the current project.

### Item 3. Who? (patients)

Who are the patients with whom wearable activity trackers will be used?

*Identify the patients (clinical group(s), characteristics, age range, mobility, socioeconomic factors, typical goals etc.), what they will be required to do (e.g. monitoring activity, charging, syncing data), and what they might need (e.g. information, instructions).*

#### Explanation

Wearable activity trackers can be useful for measuring and intervening on daily activity patterns for various patient populations [26]. In addition to identifying the population(s) of interest, outlining patient characteristics can help to develop appropriate procedures, and plan for any support that patients may require. Patients may be required to interact with the device (e.g. set goals and monitor activity) and carry out some responsibilities (e.g. charging and syncing). Devices may be complex for patients to manage, and they may require guidance and information [27].

Patient factors to consider can include age, socioeconomic factors (available resources, support and proximity to care), mobility and ability to walk safely and independently, dexterity, cognitive capacity to engage with interventions and manage devices, familiarity and confidence with technology, and goals. Identifying what patients will be required to do can highlight what specific information will be necessary for them [*see item 12 ‘Resources’*].

### Item 4. Who? (providers)

Who are the clinicians leading the use of wearable activity trackers in the service?

*Identify the lead clinicians’ role and scope, what they will be required to do (e.g. assist set-up, review data, goal setting, promote activity monitoring and engagement), and what they might need (e.g. instructions, training, dedicated time).*

#### Explanation

Healthcare professionals from varied disciplines are increasingly using wearable activity trackers in different types of services [19]. Different disciplines will vary in their scope and the type of services provided, which will influence the purpose(s) and procedures for how devices are used in different contexts. In larger interdisciplinary teams, some professions may be leading the use of wearable activity trackers and delivering services that involve wearables, while other clinicians may have a smaller role. Identifying which clinician(s) are leading their use and outlining their role and responsibilities can help meet the purpose(s) for use, and provide clarity and consistency within teams. Consider if a knowledge or skill gap exists for these clinicians, which may need to be addressed to support successful use [*see item 12 ‘Resources’*].

### Item 5. Who? (additional)

Who else is involved in supporting patients and services using wearable activity trackers?

*Identify any additional personnel, their profession (e.g. nurse, administration) or relationship (e.g. carer/family), what they will be required to do (e.g. check device is charged and worn, provide encouragement, keep track of loan devices), and what they might need (e.g. information, instructions).*

#### Explanation

Consider members in the multi-disciplinary team (e.g. nursing staff, doctors, allied health), or patient carers and relatives that may facilitate wearable activity tracker use. Additional personnel can provide support by checking devices are charged and worn, and encouraging patients. The patients’ capacity to independently manage the device and engage with care should be considered when enlisting assistance from additional personnel. For example, wearable activity trackers may provide insights on activity in patients with cognitive impairments, but their ability to independently manage the devices and engage with interventions may be limited [19]. In such circumstances, involving a carer to remind patients to wear the device and provide encouragement may be a useful strategy to support successful use [28]. If additional personnel will be involved, consider if they require any specific information or resources [*see item 12 ‘Resources’*].

### Item 6. What? (metrics)

What are the metrics of interest? (e.g. steps, daily physical activity minutes, daily sedentary behaviour minutes)

*Consider relevance to the purpose(s) and population, and accuracy for the population (including wear location).*

#### Explanation

Wearable activity trackers collect a range of metrics, which varies between models. The metrics of interest will be influenced by the purpose(s) and populations the wearable activity trackers will be used with. Identifying the metrics of interest can guide device selection. Common and relevant metrics typically include:

- Daily step count.
- Daily physical activity minutes (light, moderate-to-vigorous physical activity).
- Daily sedentary behaviour minutes.

In some circumstances, other metrics, such as sleep, oxygen saturation and heart rate, may be of interest.

The validity and accuracy of specific metrics in different populations can vary across makes and models of device [29, 30] as well as wear location on the body [31, 32]. Exploring the evidence on the validity and accuracy of metrics for different device models in the target population will help with the selection of both the metrics of interest and the specific device to be used. Ideally, metrics should provide useful information to clinicians, be relevant to patients, and be sufficiently accurate in the populations they are being used with. For example, slow-walking older adult rehabilitation patients may be suited to step count metrics, as these would correspond to goals of reducing immobility and walking more, and have demonstrated sufficient validity and accuracy in these populations [33]. Whereas younger populations with greater physical capacity and goals of exercising at higher intensities may be well suited to moderate-vigorous physical activity minutes [34].

### Item 7. What? (device characteristics)

What device(s) will be used, and what are the available characteristics?

*Consider if the device and its characteristics support the purpose(s), will meet users’ needs, and the practical considerations for ongoing use.*

#### Explanation

Characteristics to consider will typically relate to:

- Wearing the device (bodily wear site, water resistance, comfort, ease of cleaning).
- Charging the device (battery life, charging frequency (daily, weekly), time to charge).
- Device interface (feedback that is easy to understand, feedback provided on device screen vs. an application).
- Ease of set up and navigation.
- Frequency and ease of interpreting feedback provided.
- Any additional features (personalized goal setting, additional smart devices/applications that link to the wearable device).
- Software requirements.

Consider which characteristics meet the purpose and needs of users. For example, devices that are wrist-worn, simple to use, comfortable, and have attractive and discreet designs may be advantageous for patient adherence and engagement [35]. Certain characteristics may also provide additional benefits for activity interventions. For example, devices that provide real-time feedback and prompts to be active can be motivating for patients in increasing their physical activity [36].

### Item 8. How (procedures)

How will wearable activity trackers be used in the service?

*Outline the procedures to meet the intended purpose(s), support users, use devices as intended, and care for and maintain devices.*

#### Explanation

Clear procedures and planning can support successful implementation and ongoing use. Appropriate procedures can enable consistency in how devices are used, and may help to circumvent potential difficulties that may arise (such as with charging and syncing, correct wear or device loss) [19]. When planning and developing procedures, consider:

- How devices will be set up for patients.
- How metrics and data outputs will be used and documented.
- How activity intervention and promotion will be addressed (including behaviour change techniques).
- Strategies for fidelity and adherence to procedures.
- How ongoing care of devices (e.g. cleaning, charging) will be managed.
- How devices will be distributed and managed.

Procedures should correspond to the purpose(s) of use, and consider the needs of the users involved [20]. This may be achieved though undertaking a collaborative approach that involves relevant users (e.g. clinicians, patients, carers, administration) when planning and developing procedures. Pilot testing the device and the procedures prior to implementation can help to identify and address any potential problems.

### Item 9. Data access and management

How will data be accessed and managed?

*Outline data access and management, software and applications, and person(s) responsible.*

#### Explanation

Data access and management will be influenced by purpose(s) of use and the resources (human and cost) available. Methods for data access and managing data can vary in complexity and cost, and should be compatible with the setting [37]. This may be as simple as reading and recording outputs from the device interface and/or accompanying applications, or it may be more complex and involve downloading and storing raw data sets to conduct comprehensive analyses using separate software. Consider available software, and the feasibility and costs of acquiring specific software and technology (such as tablets) if required. Ensure that the data management procedures meet the privacy and security requirements of the service. Data access will involve reviewing data at different time points by specific users and personnel. When outlining data access procedures consider the method of data access, and who will be responsible for accessing and managing data.

### Item 10. When

When will measures be taken?

When will devices be provided to patients, and how long do they need to wear them for?

How often will devices need to be charged?

*Identify when to provide devices and cease their use, the frequency that data will be reviewed and devices charged, and how long patients need to wear devices for valid measures.*

#### Explanation

Key times for assessing outcomes and handling devices will need to be established. This includes: providing patients with devices, taking baseline measures, reviewing data throughout care, charging schedules, taking final measures, and ceasing use with patients based on their contact with the healthcare service. The baseline may be at a specific timepoint in treatment (e.g. intake into service), or when a specific therapy milestone is reached (e.g. ambulate independently). The device will need to be provided to patients prior to the baseline, to allow for sufficient data to be collected to form a baseline measure (e.g. the week before, or the day before assessment for monitoring periods with short durations). The device should be worn for a sufficient duration each week to provide valid measures of activity. While empirical evidence exists regarding wear time, it’s important to note that there is no universally agreed-upon recommendation that suits all applications or populations. Wear time can vary significantly depending on the specific objectives of the application, the characteristics of the population, and practical considerations around user compliance and comfort. When planning to use wearable activity trackers in healthcare, exploring the available evidence to identify a wear time that aligns with the goals and the needs of the target population is a necessary step. For instance, some studies have found that a minimum of 10 waking-hours wear time per day is effective for capturing daily physical activity measures over a shorter period [38], while up to 6 days of wear per week may be necessary for reliably monitoring activity over longer durations (i.e. several months) [39, 40]. If devices are provided to patients by the service on loan (as opposed to patients obtaining their own), the timepoint or milestone that use will be ceased should be specified in advance to prevent device loss. It is also important to consider when devices will need to be charged to determine the best time to do this around other timepoints.

### Item 11. Adaptations and modifying

Will the procedures and use of devices need to be adapted in some circumstances?

*Consider adaptations or modifications for different patients or circumstances.*

*Identify the modification and justification (e.g. different bodily wear site in very slow walkers).*

#### Explanation

In any setting, individual patient circumstances and presentations will vary. Modifications and adaptations to standard procedures may be required in some circumstances. While not all circumstances can be foreseen, planning for possible and likely modifications can support the delivery of alternate procedures when necessary. When planning or undertaking adaptations to how wearable activity trackers will be used, consider the implications of the adaptation in the context of the purpose. For example, a mixed rehabilitation service may see various patients with different ambulatory capacity. In such circumstances the wear site may vary for different patients (e.g. very slow walking patients, for whom the ankle may be a more suitable wear site for more accurate measures [33, 41]). If the purpose for using activity trackers in this example is for individual activity promotion, then varied wear sites may not be an issue. However, if the purpose and procedures involve comparing patient data, then such an adaptation may not be suitable, or the comparison methods may also require modification as the data collected from varied wear sites would not be directly comparable. Other factors to consider might include availability of carers and support, cognitive capacity for managing devices and engaging with interventions, health and digital literacy, capacity for activity, ability to safely and independently ambulate.

### Item 12. Resources

What resources are needed to support users?

*Identify what the different users involved need to support them in using wearable activity trackers in the service (e.g. information, training, software etc.).*

#### Explanation

Supporting resources and instructions will be required for users (clinicians, patients, and additional personnel) [27, 42]. Resources can include information for users on why activity is important and why wearable activity trackers are being used (the rationale), and information and instructions on how to use the devices and accompanying software (procedures). In addition to information resources, training and practical support may be required to address skill gaps [46]. When developing resources, consider what information and training different users will need, and consider collaborating with individuals who represent the different types of users to better understand their needs and preferences for resources. In some cases, documents outlining an overview of general procedures of the health service (e.g. orientation documents) may need to be updated with information about using wearable activity trackers, and direct the reader to specific supporting resources.

Patients may want to continue using wearable activity trackers following discharge or completion of contact with the healthcare service. Providing patients with information for where and how to obtain their own activity tracker (if loan devices were used) can facilitate ongoing efforts to promote physical activity and self-management.

## Applications for the WATCH

### Developing procedures for integrating and using wearable activity trackers in healthcare settings

The primary application for the WATCH is to aid the development of procedures for integrating and using wearable activity trackers to promote and monitor physical activity in different healthcare settings. It can serve as a comprehensive guide for a structured implementation process, that can help identify discrete stages and steps to be taken. This may include the selection of devices and software, developing protocols, creating relevant informational resources, providing training to involved clinicians, onboarding patients and providing them with necessary information, and the practical delivery and integration of wearable activity trackers. Having a structured approach can facilitate a smooth implementation process that is consistent with the original intent. Additional file 1 presents a fillable version of the WATCH, that allows users to compete details of their planned procedures for each item.

A feature of the WATCH is the interaction between different items and elements that it covers. Items related to the purpose, the population and the specific healthcare setting can influence other items and elements of the implementation process. For example, the metrics, device characteristics, and timepoints will be influenced by the details of the former items, and will vary for different populations and settings. We encourage a dynamic approach to using the WATCH for developing procedures, where users can work back and forth, revising details for each item as they use it.

A key part of developing an implementation plan is the selection of which wearable activity tracker device will be used. Outlining a plan using the WATCH can help users to make an informed selection of which device and software can meet the requirements of the purpose and setting. Available devices and software are varied in their features and capabilities, and they are continually changing as technology rapidly evolves. Given the current absence of an ‘ideal’ device and software platform for use in healthcare settings [46], the WATCH can play an important role in helping users to identify their needs, and adapt how they use available devices and software to meet their purposes.

The WATCH may also be used to facilitate a stakeholder involvement process by identifying the individuals who can play important roles in the implementation process. This can prompt collaboration and engagement among stakeholders before and during implementation [43]. Identifying these stakeholders and outlining their roles for implementation can lead to effective and clear communication about the implementation process, provide opportunities to gain valuable perspectives, and identify potential skill and knowledge gaps to be addressed [44]. This then informs the critical stage of preparing and training personnel who will be at the frontline of integrating and using wearable activity trackers in service delivery. Involvement and consultation of stakeholders ahead of implementation can identify the training and information required for the various individuals and how to best deliver this.

By outlining the procedures and stages in advance, users can ensure that the planned implementation of wearable activity trackers is also consistent with the quality standards and requirements of the specific healthcare setting or service. This could include professional standards, duty of care and scope of practice of the clinicians involved, as well as integration into existing workflows and methods of care delivery, and integration into existing technology ecosystems that are available [26].

### Using the WATCH for evaluation

During and following implementation of the planned approach to using wearable activity trackers, the WATCH may be used to evaluate the success of delivery. It can facilitate assessment if the actions carried out during implementation are consistent to those outlined from the outset. This aids in identifying challenges or problems impacting implementation, as well as potential solutions or strategies for future efforts. Likewise, the WATCH can highlight achievements and factors contributing to successful implementation. This valuable information can help to define performance indicators essential for maintaining sustained use and for optimising patient outcomes. Additional file 2 presents an evaluation-focussed version of the checklist, that allows users to record details of their implementation efforts against their planned procedures.

Using the WATCH for evaluation allows for the identification of key factors for success that may have been overlooked during the development stage, or determine if certain factors were irrelevant. Evaluation may encompass a review of a pilot phase to understand the requirements for scaling the initiative. Beyond this initial phase, ongoing evaluation can provide vital information for longer-term sustainability, and for planning larger-scale expansion within the specific healthcare setting.

### Adaptability and scaling

Integrating wearable activity trackers in healthcare settings is complex. Implementation involves multiple stakeholders, requires behavioural changes from both providers and recipients, and has the potential to influence diverse outcomes [45]. Moreover, healthcare systems themselves are complex and multi-faceted, with various contextual factors and stakeholder interactions shaping their operation and the integration of new innovations. Because of these complexities, tailoring wearable activity tracker use for specific healthcare settings is crucial to ensure that procedures align well with different contexts, thereby increasing the likelihood of achieving intended outcomes [22].

This resource recognises that healthcare environments and services are varied and come with their own unique set of requirements. Likewise, the successful implementation of wearable activity trackers will differ across these diverse settings. A key strength of the WATCH lies in its ability to adapt to the distinct needs of each healthcare context. Such adaptability is not just beneficial, but essential for achieving scalability across a range of healthcare settings.

## Benefits of the WATCH

The WATCH presented in this paper supports progress toward the implementation of wearable activity trackers in healthcare settings for physical activity promotion and monitoring. A key benefit is its versatility. It provides a comprehensive yet adaptable framework that can guide a wide spectrum of users in establishing structured and co-ordinated approaches for integrating wearable activity trackers across various healthcare settings. This is particularly beneficial for newcomers who may have limited experience and knowledge in using wearable activity trackers, and are unsure about what clinical considerations are essential. Another important benefit is the WATCH’s capacity to not only guide the development, but also evaluate implementation efforts. This can identify key factors leading to challenges and successes of implementation, highlighting important areas for development or maintenance for successful and effective use. Overall, the applications and benefits of the WATCH can support a more informed and systematic approach for integrating wearable activity trackers in a range of healthcare settings, ultimately enhancing healthcare delivery and improving a broad array of outcomes associated with increasing physical activity levels.

## Future directions

To our knowledge, this checklist is the first of its kind. An important next step will be in pilot testing and evaluating the clinical utility of the WATCH for developing wearable activity tracker implementation procedures and evaluating such efforts. The use of wearable activity trackers and associated technology in healthcare is rapidly advancing, and the types of devices and software available to healthcare providers will continue to grow over time. As such, periodic updates to the WATCH and associated user resources will likely become necessary as the healthcare oriented wearable activity trackers market expands.

The WATCH is primarily focussed on the integration of wearable activity trackers in healthcare settings for physical activity promotion and monitoring purposes. It was developed based on insights gained through a Delphi study that explored wearable activity tracker use for purposes related to these applications. It is important to note that different approaches may be required for other wearable sensor technologies used for distinct forms of medical monitoring, such as continuous glucose monitoring or Holter monitoring.

## Conclusion

In summary, the WATCH serves as an innovative tool designed to guide the complex process of integrating wearable activity trackers into various healthcare settings for physical activity promotion and monitoring. We hope that its versatility and adaptability will make it useful for practitioners from diverse healthcare professions and settings, ranging from novices to experts, by providing a structured yet adaptable framework for implementation and evaluation. Future work will focus on assessing its clinical utility and adaptability to other wearable technologies, and as technology rapidly evolves, the WATCH will require updates to remain relevant and helpful. This work aims to facilitate a more informed, systematic, and ultimately successful integration of wearable activity trackers into healthcare settings, with the goal of enhancing patient outcomes through increased levels of physical activity.

### Electronic supplementary material

Below is the link to the electronic supplementary material.

**Additional file 1**: Fillable planning checklist

**Additional file 2**: Fillable evaluation checklist

## Data Availability

The Additional files for this manuscript contain planning and evaluation versions of The Wearable Activity Tracker Checklist for Healthcare (WATCH) for users.

## Abbreviations

WATCH: Wearable Activity Tracker Checklist for Healthcare
CFIR: Consolidated Framework for Implementation Research
