# Supplementary material for: The Wearable Activity Tracker Checklist for Healthcare (WATCH): a 12-point guide for the implementation of wearable activity trackers in healthcare
Source: Int J Behav Nutr Phys Act. 2024 Mar 13;21:30. doi: 10.1186/s12966-024-01567-w (PMC10938760; doi:10.1186/s12966-024-01567-w)
Supplement: Supplementary file 2 — Additional file 2: Fillable evaluation checklist [file 12966_2024_1567_MOESM2_ESM.pdf]

The Wearable Activity Tracker Checklist for Healthcare (WATCH)

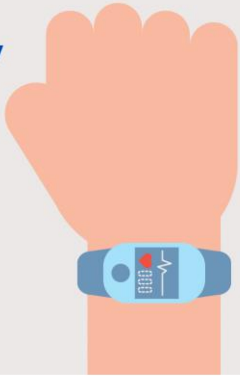

Project Evaluation

Details

**Project Name**	
**Project Leader**	
**Start Date**	
**Evaluation Date**	

Checklist

Item	What was planned?	What happened?
**1. Why**<br><br>Why are wearable activity trackers being used in the service?<br><br>*Identify the purpose(s) and goal(s).*		

**2. Where**<br><br>In what setting/clinical context will you be using wearable activity trackers?<br><br>*Identify the health service setting, including location(s), nature of patient contact, and relevant infrastructure (e.g. internet connection, safe walking spaces).*<br><br>*Identify if previous efforts have been made to implement wearable activity trackers in the service.*		
**3. Who (patients)**<br><br>Who are the patients with whom wearable activity trackers will be used?<br><br>*Identify:*<br><br>*- The patients (clinical characteristics, age range, mobility/ambulation, socioeconomic factors, goals etc.)*<br><br>*- What they will be required to do (e.g. monitoring activity, charging device, syncing data)*<br><br>*- What they might need (e.g. information and instructions).*		
**4. Who (providers)**<br><br>Who are the clinicians leading the use of wearable activity trackers in the service?<br><br>*Identify the lead clinicians:*<br><br>*- Role and scope, what they will be required to do (e.g. set-up, review data, goal setting, promote activity monitoring)*<br><br>*What they might need (e.g. instructions and training, dedicated time).*		

**5. Who (additional)**<br><br>Who else is involved in supporting patients and services using wearable activity trackers?<br><br>*Identify any additional personnel:*<br><br>- *profession or relationship to patient (e.g. nurse, administration, carer/family)*<br><br>- *what they will be required to do (e.g. check device is charged and worn, provide encouragement, keep track of loan devices)*<br><br>- *What they might need (e.g. information and instructions).*		
**6. What (metrics)**<br><br>What are the metrics of interest? (e.g. steps, daily minutes of physical activity, daily minutes of sedentary behaviour)<br><br>*Consider relevance to the purpose(s) and population, and accuracy for the population (including wear location).*		
**7. What (device characteristics)**<br><br>What device(s) will be used, and what are the available characteristics?<br><br>*Consider if the device and its characteristics support the purpose(s), will meet users' needs, and the practical considerations for ongoing use.*		

**8. How (procedures)**<br><br>How will wearable activity trackers be used in the service?<br><br>*Outline the procedures to meet the intended purpose(s), support users, use devices as intended, and care for and maintain devices.*		
**9. Data access and management**<br><br>How will data be accessed and managed?<br><br>*Outline data access and management, software and applications, and person(s) responsible.*		
**10. When**<br><br>When will measures be taken?<br><br>When will devices be provided to patients, and how long do they need to wear them for?<br><br>How often will devices need to be charged?<br><br>*Identify timepoints for providing devices and ceasing use, the frequency/time points that data will be reviewed, charging, and how long patients need to wear devices for valid measures.*		

**11. Adaptations and modifying**<br><br>Will the procedures and use of devices need to be adapted in some circumstances?<br><br>- *Consider adaptations or modifications for different patients or circumstances.*<br><br>*Identify the modification and justification (e.g. different bodily wear site in very slow walkers).*		
**12. Resources**<br><br>What resources are needed to support users?<br><br>*Identify what the different users involved need to support them in using wearable activity trackers in the service (e.g. information, training, software etc.).*		
